# Characterization of unusual iAMP21 B‐lymphoblastic leukemia (iAMP21‐ALL) from the Mayo Clinic and Children's Oncology Group

**DOI:** 10.1002/gcc.23084

**Published:** 2022-07-19

**Authors:** Alaa Koleilat, James B. Smadbeck, Cinthya J. Zepeda‐Mendoza, Cynthia M. Williamson, Beth A. Pitel, Crystal L. Golden, Xinjie Xu, Patricia T. Greipp, Rhett P. Ketterling, Nicole L. Hoppman, Jess F. Peterson, Christine J. Harrison, Yassmine M. N. Akkari, Karen D. Tsuchiya, Mary Shago, Linda B. Baughn

**Affiliations:** ^1^ Division of Laboratory Genetics and Genomics, Department of Laboratory Medicine and Pathology Mayo Clinic Rochester Minnesota USA; ^2^ Division of Computational Biology, Department of Quantitative Health Sciences Mayo Clinic Rochester Minnesota USA; ^3^ ARUP Laboratories Salt Lake City Utah USA; ^4^ Division of Hematopathology, Department of Laboratory Medicine and Pathology Mayo Clinic Rochester Minnesota USA; ^5^ Leukaemia Research Cytogenetics Group, Translational and Clinical Research Institute Newcastle University Centre for Cancer Newcastle‐upon‐Tyne UK; ^6^ Cytogenetics and Molecular Pathology, Legacy Health Portland Oregon USA; ^7^ Department of Laboratory Medicine and Pathology University of Washington Seattle WA USA; ^8^ Department of Paediatric Laboratory Medicine, The Hospital for Sick Children University of Toronto Toronto Ontario Canada

**Keywords:** b‐all, cytogenetics, fish, iamp21‐all, runx1

## Abstract

Acute lymphoblastic leukemia (B‐ALL) with intrachromosomal amplification of chromosome 21 (iAMP21‐ALL) represents a recurrent high‐risk cytogenetic abnormality and accurate identification is critical for appropriate clinical management. Identification of iAMP21‐ALL has historically relied on fluorescence in situ hybridization (FISH) using a *RUNX1* probe. Current classification requires ≥ five copies of *RUNX1* per cell and ≥ three additional copies of *RUNX1* on a single abnormal iAMP21‐chromosome. We sought to evaluate the performance of the *RUNX1* probe in the identification of iAMP21‐ALL. This study was a retrospective evaluation of iAMP21‐ALL in the Mayo Clinic and Children's Oncology Group cohorts. Of 207 cases of iAMP21‐ALL, 188 (91%) were classified as “typical” iAMP21‐ALL, while 19 (9%) cases were classified as “unusual” iAMP21‐ALL. The “unusual” iAMP21 cases did not meet the current definition of iAMP21 by FISH but were confirmed to have iAMP21 by chromosomal microarray. Half of the “unusual” iAMP21‐ALL cases had less than five *RUNX1* signals, while the remainder had ≥ five *RUNX1* signals with some located apart from the abnormal iAMP21‐chromosome. Nine percent of iAMP21‐ALL cases fail to meet the FISH definition of iAMP21‐ALL demonstrating that laboratories are at risk of misidentification of iAMP21‐ALL when relying only on the *RUNX1* FISH probe. Incorporation of chromosomal microarray testing circumvents these risks.

## 1 INTRODUCTION

Acute lymphoblastic leukemia (B‐ALL), involving B‐lineage precursor cells, is the most common childhood malignancy with an estimated 5700 new cases (adult and pediatric) in the United States in 2021.^1^ Cytogenetic classifications of B‐ALL is an important component of patient risk stratification and clinical management.^2^ B‐ALL with intrachromosomal amplification of chromosome 21 (iAMP21‐ALL) is a distinct high‐risk entity accounting for approximately 2% of B‐ALL. It is associated with a median age of 9 years and a low white blood cell count (median 5 × 10^9^/L).^3^, ^4^ Formation of the iAMP21‐chromosome has been shown to arise from breakage‐fusion‐bridge cycles, resulting in chromothripsis of chromosome 21 with a variable region of copy number gain often, but not always, including the *RUNX1* gene (at 21q22).^5^, ^6^ Deletion of the telomeric region of 21q, between genomic positions 44 and 47 Mb, has also been frequently observed.^5^, ^7^, ^8^, ^9^ As the *RUNX1* gene is often found in the highest region of chromosome 21 gain, fluorescence in situ hybridization (FISH) probes directed to the *RUNX1* gene as part of the *ETV6*::*RUNX1* fusion probe set is an efficient and cost‐effective method to identify iAMP21‐ALL within clinical cytogenetics laboratories. Currently, iAMP21‐ALL is defined as greater than or equal to three extra copies of the *RUNX1* gene on a single abnormal chromosome 21 (iAMP21‐chromosome) and greater than or equal to five copies of the *RUNX1* gene region per interphase cell.^3^ However, accurate identification of iAMP21‐ALL can be challenging as confirmation often requires FISH of abnormal metaphase cells, which are not always available, to distinguish polysomy 21 associated with favorable high hyperdiploidy from iAMP21‐ALL. Metaphase FISH also allows the determination of the chromosomal location of the additional *RUNX1* signals, which are typically restricted to a single iAMP21‐chromosome. However, rare, unusual cases of iAMP21‐ALL have been previously described in which the extra *RUNX1* signals are located on more than one abnormal chromosome.^10^, ^11^ While these unusual iAMP21‐ALL cases did not fit the current FISH definition of iAMP21, chromosomal microarray analysis (CMA) confirmed the characteristic chromosome 21 copy number profile seen in iAMP21‐ALL. As FISH is the most common, rapid, and cost‐effective method used in the genomic characterization of B‐ALL, we evaluated the performance of the *RUNX1* probe in the identification of iAMP21‐ALL and determined the frequency of unusual iAMP21‐ALL in two large cohorts of B‐ALL. Here, we describe 14 cases of B‐ALL with genomic features of iAMP21‐ALL by CMA that failed to meet the current definition of iAMP21‐ALL using FISH. We discuss an algorithmic approach to the genomic evaluation of B‐ALL to reduce the misidentification and subsequent risk of misclassification of these unusual iAMP21‐ALL cases.

## 2 MATERIALS AND METHODS

### 2.1 Patient selection

This study represented an institutional review board (IRB) approved retrospective evaluation of iAMP21‐ALL from unique patients in two cohorts, Mayo Clinic and Children's Oncology Group (COG). See study design and participant selection (Figure S1). For the Mayo Clinic cohort, we performed a search of all cases of pediatric B‐ALL from patients (≤30 years of age) with evidence of clinical trial enrollment (*n* = 777) and all B‐ALL from patients with evidence of iAMP21‐ALL without clinical trial enrollment from January 2018 to December 2020. For the COG cohort, we performed a search of all patients with B‐ALL that were enrolled on the COG APEC14B1 study (Project: Every Child: a registry, eligibility screening, biology, and outcome study) between August 2018 and June 2021. Cases with genetic evidence of iAMP21‐ALL and with CMA were further analyzed. Data were obtained from the COG registry and no additional experiments were performed on the COG cases. The methods below are specific for the Mayo Clinic cases.

### 2.2 Chromosome analysis

White blood cells from the diagnostic bone marrow (BM) aspirate or lymph node specimen were cultured (24‐ and 48‐h, unstimulated), harvested and G‐banded slides were prepared using standard cytogenetic techniques. Where possible 20 metaphases were analyzed and two karyograms prepared from each patient.^12^ Chromosome analysis of BM specimens was performed in COG‐approved local laboratories. Karyotypes were noted to represent unusual iAMP21‐ALL at the time of central review.

### 2.3 Fluorescence in situ hybridization

All fluorescence in situ hybridization (FISH) probes were supplied by Abbott Molecular (AM) (Des Plaines, IL) unless otherwise specified as a laboratory‐developed test (LDT), Cytocell (CC) (Cambridge), or Agilent Technologies (AT). FISH studies for B‐ALL were routinely performed in the Mayo Clinic Genomics Laboratory using 11 probe sets: *PBX1*::*TCF3* for *t*(1;19)(q23;p13.3) (LDT), D4Z1/D10Z1/D17Z1 for +4/+10/+17 (AM), *3′MYC*/*5′MYC* for 8q24 (AM), *CDKN2A*/D9Z1 for 9p−/+9 (AM), *ABL1*::*BCR* for *t*(9;22)(q34;q11.2) (AM), 5*′KMT2A*/3*′KMT2A* for 11q23 (LDT), *ETV6*::*RUNX1* for *t*(12;21)(p13;q22) (LDT), *3′IGH*/*5′IGH* for 14q32: *IGH* (LDT), *TP53*/D17Z1 for −17/17p‐ (AM), *3′CRLF2*/*5′CRLF2* for Xp22.33/Yp11.32:*CRLF2* (CC), *3′P2RY8*/*5′P2RY8* for Xp22.33/Yp11.32:*P2RY8* (CC). In addition, Ph‐like ALL FISH panel was utilized which includes 5 probe sets: *3′ABL2*/*5’ABL2* for 1q25 (LDT), *3′PDGFRB*/*5′PDGFRB* for 5q33 (LDT), *IKZF1/*Cep 7 for 7p−/−7 (AT), *5′JAK2*/*3′JAK2* for 9p24.1 (LDT), and *5′ABL1*/*3′ABL1* for 9q34 (LDT). Cells were subjected to standard FISH pretreatment, hybridization, and fluorescence microscopy. FISH analysis of BM specimens was performed in COG‐approved local laboratories.

### 2.4 Chromosomal microarray analysis

SNP CMA was performed using the ThermoFisher Scientific CytoScan™ HD array platform (Waltham, MA) using an input of up to 250 ng DNA. Samples were digested with a restriction enzyme, ligated to adapters, amplified by PCR, fragmented, labeled with biotin, and hybridized to a CytoScanHD™ array chip overnight. The chip was then washed and stained in a GeneChip® Fluidics Station 450 and scanned in a GeneChip® Scanner 3000. The resulting data were visualized and analyzed using chromosome analysis suite (ChAS) 3.3. CMA of BM specimens was performed in COG‐approved local laboratories.

### 2.5 Mate pair sequencing

DNA was processed using the Illumina Nextera Mate Pair library preparation kit (Illumina, San Diego, CA) and sequenced on the Illumina HiSeq 2500 using 101‐basepair reads and paired‐end sequencing. Data were aligned to the reference genome (GRCh38) using BIMAv3^13^ and abnormalities were identified and visualized using in‐house developed bioinformatics pipeline, BMD‐SV Pipeline.^14^, ^15^, ^16^ Structural variants involving chromosome 21 were plotted using Circos.^17^

### 2.6 Statistical analysis

A Fisher's exact test was used to determine the significance between the number of males and females between the two iAMP21‐ALL groups and a two‐sided unpaired *t*‐test was performed to determine the significance between the number of *RUNX1* signals from the typical and unusual iAMP21‐ALL cases (https://www.graphpad.com/quickcalcs/).

## 3 RESULTS

### 3.1 Frequency of unusual iAMP21‐ALL

**FIGURE 1:**
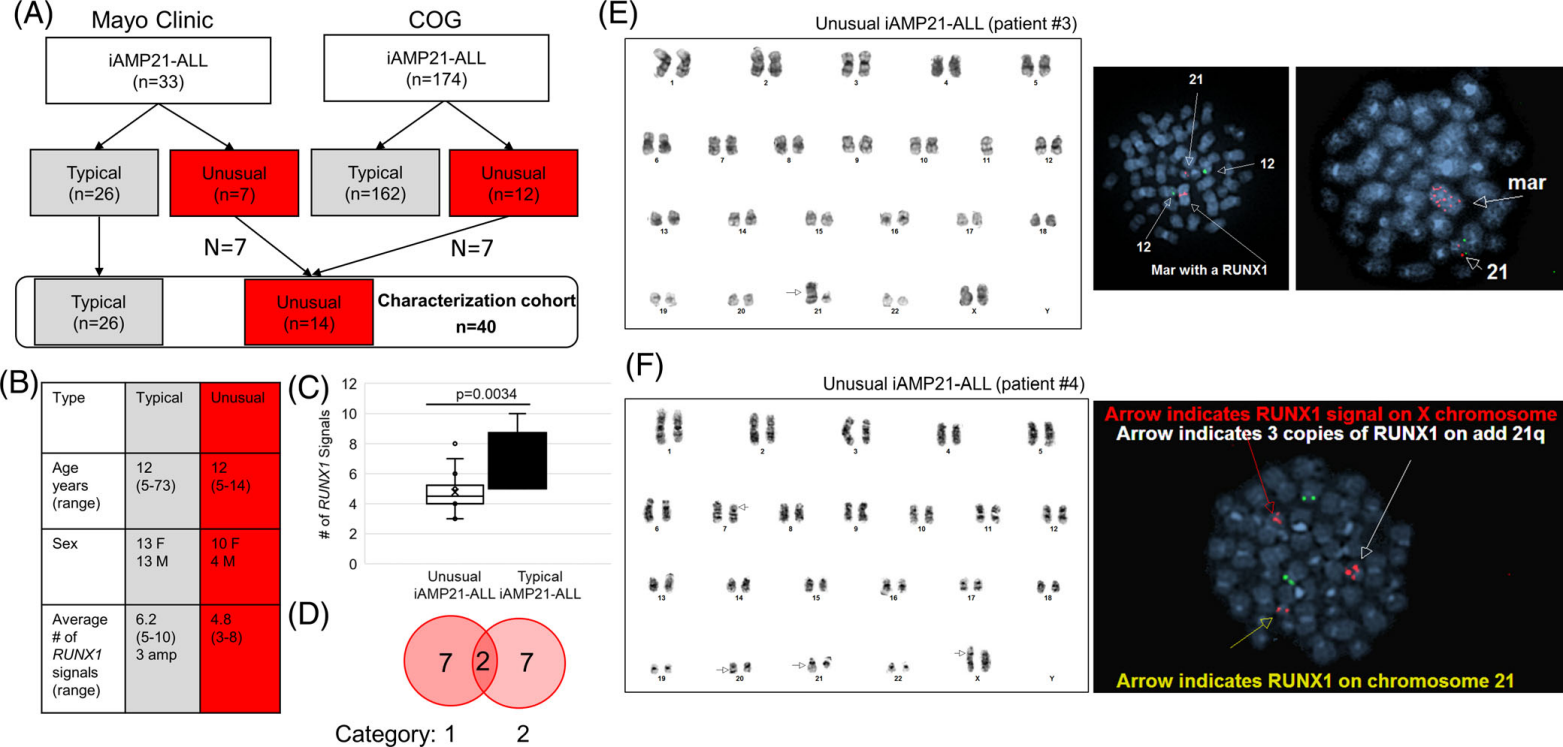
Cohort description of typical and unusual iAMP21‐ALL cases. (A) iAMP21‐ALL cohort. (B) Summary of the demographic and average number (#) *RUNX1* signals in typical and unusual iAMP21‐ALL cases. (C). Box and whisker plot displaying the distribution of *RUNX1* signals in the unusual and typical iAMP21‐ALL subgroups. A two‐sided Student's *t*‐test was performed to determine the statistical significance. (D) Venn diagram demonstrating number of unusual cases that fit into Category 1, Category 2, or both. Category 1 is defined those with fewer than 5 *RUNX1* signals per cell. Category 2 is defined as those with the additional *RUNX1* signals located apart from the abnormal iAMP21‐chromosome. (E) Patient case #3 of unusual iAMP21‐ALL showing an abnormal chromosome 21 by conventional chromosome analysis and metaphase FISH using the *ETV6::RUNX1* probe demonstrated 3 copies of *RUNX1* (21q22.12), with 2 signals on a marker chromosome, later clarified as the iAMP21‐chromosome and metaphase FISH using probes CTD‐2226O4 targeting 21q22.13 (multiple red signals on the abnormal chromosome 21) and CTD‐2119D4 (green) targeting the distal end of chromosome 21q22.3 confirmed iAMP21‐ALL. (F) Patient case #4 of unusual iAMP21‐ALL showing an abnormal chromosome 21 and an abnormal chromosome X by conventional chromosome analysis. Metaphase FISH using the *ETV6*::*RUNX1* probe demonstrated 5 copies of *RUNX1* with additional copies of *RUNX1* on the abnormal X chromosome in addition to an abnormal chromosome 21

To determine the frequency of unusual iAMP21‐ALL, we performed a retrospective evaluation of the Mayo Clinic and the COG databases for cases classified as iAMP21‐ALL. In total, 207 non‐overlapping cases of iAMP21‐ALL were identified using the *RUNX1* FISH probe. Using the current definition of iAMP21‐ALL (greater than or equal to three extra copies of *RUNX1* on a single abnormal chromosome 21 and greater than or equal to five copies of the *RUNX1* gene region per cell), 188 (91%) cases met this definition and were classified as “typical” iAMP21‐ALL, while 19 (9%) cases failed to meet this definition and were classified as “unusual” iAMP21‐ALL (Figure 1A, Figure S1 **)**. Of these 19 unusual cases, five cases were removed from our cohort due to the lack of residual specimens for further characterization. Chromosomal microarray (CMA) was used to confirm iAMP21‐ALL in cases that did not meet the original definition of iAMP21‐ALL. Overall, of the 207 iAMP21‐ALL cases, 40 cases were further characterized and 26 typical and 14 unusual iAMP21‐ALL were compared. Although patients with unusual iAMP21‐ALL had a similar median age at diagnosis (12 years, range 5–14 years) compared to patients with typical iAMP21‐ALL (12 years, range 5–73 years), there was a higher proportion of female patients with unusual iAMP21‐ALL (71%) compared to typical iAMP21‐ALL (50%) (*p* = 0.3152) (Figure 1, Table 1). In addition, of the 40 cases utilized in this study, two cases demonstrated a Robertsonian *t*(21;22)(q10;q10) (patient 6) and *t*(15;21)(q10;q10) (patient 7) (Table 1).

**TABLE 1:** Summary of iAMP21‐ALL cases

Patient	Sex	Age (years)	Conventional chromosomal analysis	Typical or unusual iAMP21‐ALL	Max # of *RUNX1* signals by interphase FISH^a^
1	F	12	45,XX,add(1)(p13),−2,der(6)*t*(6;8)(p25;q13),der(7)t(7;11)(p22;q13),−8,−11,add(16)(p11.2),+2mar [1]/ 45,XX,−1,dic(1;3)(p22;p23),add(5)(p15.1),−18,−19,+3mar [1]/46,XX [18]	Unusual‐2	5
2	M	14	45,X,−Y,der(9;17)(q10;q10),dic(19;?)(p13.3;?),+21,r(21)x2 [2]/45,X,−Y,der(9;17)(q10;q10),dic(19;?)(p13.3;?),dic(21;21)(q22;q22)ins(21;?)(q22;?) [1]/ 46,XY,+1,der(9;17)(q10;q10),add(10)(q26),der(21)ins(21)(q11.2)i(21)(q10)inv(21)(q11.2;q22) [7]/ 44,−Y [1],der(X)t(X;11)(p22;q13),add(2)(p13),i(8)(q10),der(9;17)(q10;q10),dic(11;19)(q11;p13.3), r(14),der(21)i(21)(q10)inv(21)(q11.2q22) [1],inv(21)(q11.2q22) [1],der(21)add(21)(p11.2) [1], der(21)dup(21)(q11.2q22)add(21)(q22) [2][cp6]/46,XY [4]	Unusual‐1 + 2	4
3	F	8	45,XX,der(9)*t*(9;11)(p13;q11)del(11)(q13q25),−11,der(21)(21q21–>21q22::21q22–>21q21::21q22–>21q11.2::21q11.2–>21q22) [6]/46,sl,t(5;19)(p11;q13.3),−15,+2mar [2]/44,XX,der(9)t(9;11)(p13;q11)del(11)(q13q25),−11,der(21)(21q21–>21q22::21q22–>21q21::10pter–>10qter::21q22–>21q11.2::21q11.2–>21q22) [3]/44,sl2,*t*(4;16)(p16;q24) [2]/46,XX [7]	Unusual‐1	3
4	F	10	46,X,der(X)t(X;21)(p22.1;q22),*t*(7;20)(p15;q13.3),add(21)(q22) [13]/46,XX [7]	Unusual‐2	5
5	F	8	47,XX,+X,−5,add(13)(q32),add(21)(q22),+mar [15]//46,XX [4]	Unusual‐2	5
6	F	5	47,XX,der(21;22)(q10;q10)?c,+2r[3]/45,XX,der(21;22)(q10;q10)?c[8]	Unusual‐2	8
7	F	12	47,XX,+X,+10,−15,?add(17)(q25),−21,+mar/45,XX,der(15;21)(q10;q10)?c	Unusual‐1	4
8	F	12	48,XX,der(7)*t*(X;7)(p11.2;p22),del(12)(p12p13),+21,+21,der(21)dup(21)(q21q22)del(21)(q22)x3 [18]/46,XX [2]	Unusual‐2	7
9	M	11	47,XY,+X,*t*(2;21)(p11.2;q22),add(9)(p13),add(21)(q22) [11]/46,XY [9]	Unusual‐1	4
10	M	6	46,XY,add(7)(q22),−13,add(13)(p11.1),−16,add(21)(q22),+mar1,+mar2 [10]/46,XY [6]	Unusual‐1	3
11	F	12	46,XX,add(8)(p21),der(21)?r(21;?)(p11.2q22;?)[13]/46,XX [7]	Unusual‐1	4
12	M	7	48,XY,+21,+add(21)(q22) [2]/46,XY [18]	Unusual‐2	6
13	F	12	46,X,add(X)(q22),add(10)(p13),add(16)(q11.2),del(17)(p11.2) [9]/45,sl,?der(8;22)(q10;q10),add(18)(q21.1) [11]/46,XX [3]	Unusual‐2	5
14	F	13	57,XX,+X,+4,+5,+add(6)(q23),add(7)(p11.2),+10,+11,add(12)(p11.2),+14,del(16)(q12),+17,+18,+add(21)(q11.2),+mar[19]/ 46,XX [1]	Unusual‐1 + 2	4
15	F	10	46,XX,i(21)(q10)dup(21)(q22q21) [10]/46,XX [10]	Typical	5
16	M	5	45,XY,−19,add(20)((q11.1),der(21)(21q21–>21q21)::(21q21–>21q22)::(21q22–>21q10)::(21q10–>21q22)::(21q22–>21q21)::(21q21–>21q21) [15]/46,XY [5]	Typical	5
17	F	17	43–46,XX,dup(1)(q21q42),add(3)(q21),der(7)add(7)(p11.2)add(7)(q32),−10,−13,add(21)(q22),+add(22)(q13),+der(?)t(?;3); (?;q21),+0–1mar[cp3]/46,XX [17]	Typical	5
18	F	28	46,XX,*t*(1;11)(p13;p15),*t*(2;9)(p15;q32),del(6)(q13),add(7)(p22),del(13)(q14q22),−21,+0–1r,+0–2mar[cp4]/46,XX [16]	Typical	8
19	F	10	46,XX,del(7)(q22q34),−8,add(9)(q22),−13,add(14)(q24),+r,+1–2mar[cp10]/46,XX [10]	Typical	5
20	M	24	44–47,XY,der(2)t(2;5)(p21;q13),hsr(21)(q22)[cp2]/46,XY [9];	Typical	9
21	M	10	46,XY,mar(21)(q22) [6]/46,XY [14]	Typical	5
22	F	13	46,XX,der(21)(pter‐>q22::q22–>q21::q21–>q22::q22–>q21::q22–>qter) [3]/46,X,−X,del(9)(p13p22),der(21)(pter–>q22::q22‐>q21::q21‐ > q22::q22–>q21::q22–>qter),+mar[11]/46,XX [7]	Typical	5
23	M	16	Not available	Typical	Amp
24	M	13	46,XY [20]	Typical	8
25	M	9	46,XY,ider(21)(q10)dup(21)(q10q22) [9]/46,XY [11]	Typical	9
26	M	11	46,XY,der(21)ins(21;?)(p11.2;?)add(21)(q22) [16]/46,XY [4]	Typical	5
27	F	14	47,XX,+X,t(7;10)(p15;q22),add(9)(p13),add(9)(p13),del(13)(q12q22),der(21)add(21)(p11.2)add(21)(q22) [8]/46,XX [9]	Typical	Amp
28	F	73^b^	88–90,XX,−X,−X,−1,−1,add(1)(q12),add(1)(q12),add(3)(p25)x2,+6,−7,−7,−8,−10,+11,der(11)t(1;11)(q21;q23)x2,add(12)(p11.2), add(12)(p11.2),+14,+add(14)(q32)x2,+15,+18,−21[cp2]/46,XX [11]	Typical	10
29	F	12	46,XX,add(21)(q22) [20]	Typical	6
30	M	8	47,XX,+X,r(21)(p11.1q22) [1]/47,idem,r(19)(p13.3q13.4) [19]	Typical	5
31	F	12	46,XX,der(16)t(1;16)(q21;q12.1),r(21) [3]/46,XX [8]	Typical	5
32	F	7	47,XX,der(7)t(7;9)(p15;q13),der(9)t(7;9)del(9)(p13),add(10)(q24),del(11)(q13),del(13)(q14q32),del(15)(q12q15),add(16) (q12),der(21)(21qter–>21c::21c–>21q22::21q11.2‐>21qter),+22[19]/46,XX[1]	Typical	5
33	M	8	46,XY,der(10)add(10)(p11.2)add(10)(q24),−13,add(20)(q11.2),add(21)(q22),+mar [9]/46,XY [6]	Typical	7
34	F	9	46,XX,inv(10)(p11.2q22)?c,del(20)(q13.1),ider(21)(q22),add(22)(p11.1)[13]/ 46,XX,add(2)(q23),add(7)(q32),inv(10)(p11.2q22)?c,add(12)(p13),del(20)(q13.1),ider(21)(q22) [2]/ 46,XX,inv(10)(p11.2q22)?c [7]	Typical	9
35	M	15	46,XY,del(12)(p11.2),der(12)t(12;13)(q24.3;q14),−13,+mar [5]/46,sl,add(21)(q22)[cp5]/46,XY [10]	Typical	6
36	M	14	46,XY,add(10)(q22),add(21)(q22) [3]/46,XY [17]	Typical	5
37	F	12	46,XX,del(4)(q12q21),add(15)(q22),add(21)(q22) [6]/46,XX [14]	Typical	Amp
38	M	8	47,XY,+X,ins(5;?)(q11.2;?),del(16)(q11.1),add(21)(q22) [7]/47,sl,add(1)(q32) [7]/46,XY[6]	Typical	6
39	F	11	46,XX,add(7)(q22),add(21)(q22) [7]/46,XX [13]	Typical	5
40	M	18	46,XY,add(9)(p13),add(9)(p13),der(21)(21pter‐>21q22.3::21q22.1–>21q22.3::21q22.3–>21q22.1::21q22.3–>21p11.1) [8]/46,sl,t(10;19)(q24;q13.1)[10]/46,XY[2]	Typical	5
*n* = 26	13 F:13 M Median age 12 (5–73) years			Typical	5 (5‐amp)
*n* = 14	10 F: 4 M Median age 12 (range 5–14) years			Unusual	5 (3–8)

^a^ If number of *RUNX1* signals observed by interphase FISH is indicated as “amplification” that means that there were greater than 10 signals per interphase cell.

^b^ Confirmed with pathology report that this patient has B‐ALL.

### 3.2 Unusual iAMP21‐ALL does not meet the current definition of iAMP21‐ALL by FISH

The total number of *RUNX1* signals could be accurately quantified by interphase FISH in 37 (93%) iAMP21‐ALL cases, with a median number of five *RUNX1* signals (range 3–10) per cell. However, in three (7%) cases, the total number of *RUNX1* signals could not be accurately quantified due to their large number of *RUNX1* signals (>10) per cell; thus, these cases were indicated simply as “*RUNX1* amplification.” The average maximum number and range of *RUNX1* signals by interphase FISH were significantly higher in cases with typical iAMP21‐ALL compared to unusual iAMP21‐ALL (typical: average 6.2, range 5–10, unusual: median 4.8, range 3–8) (*p* < 0.0034) (Figure 1B, C). All 26 typical iAMP21‐ALL cases met the current definition of iAMP21‐ALL, which required a total of five or more *RUNX1* signals per cell by interphase FISH or three or more extra *RUNX1* signals on the iAMP21‐chromosome by metaphase FISH (Table 1, data not shown). In contrast, none of the 14 unusual iAMP21‐ALL cases met the current definition of iAMP21‐ALL (Tables 1 and 2). Of these 14 unusual iAMP21‐ALL cases, 7 (50%) cases (patients 2, 3, 7, 9, 10, 11, and 14) had fewer than 5 total *RUNX1* signals per cell by interphase FISH (“Category 1”) (Figure 1D, E, Tables 1 and 2). While the remaining 7 unusual iAMP21‐ALL (50%) cases (patients 1, 4, 5, 6, 8, 12, and 13) had five or more *RUNX1* signals per cell by interphase FISH, the additional *RUNX1* signals were located apart from the abnormal iAMP21‐chromosome (“category 2”) (Figure 1D, F, Tables 1 and 2). Two patients (2 and 14) had less than 5 total copies of *RUNX1* signals per cell and also demonstrated *RUNX1* signals on chromosomes other than the abnormal iAMP21‐chromosome (“Categories 1 and 2”) (Figure 1D, Tables 1 and 2). The *RUNX1* signals were found on other chromosomes in addition to chromosome 21 in 6 (43% of unusual iAMP21‐ALL) cases (patients 1, 2, 4, 5, 6, and 14). These additional chromosomes included chromosomes 10 and 20 in patient 1, a ring chromosome 8 in patient 2, chromosome X in patient 4, a derivative chromosome involving chromosomes 5 and 13 in patient 5, chromosomes 18, 22, and ring chromosome 13 in patient 6 and chromosome 7 in patient 14 (Table 2).

**TABLE 2:** Cytogenetics summary of unusual iAMP21‐ALL cases

Patient	Maximum # of *RUNX1* signals by FISH		Chromosomes with *RUNX1* signals	Mate pair	Max copy state of chromosome 21	# of copy state changes	Copy # fluctuations (low and high)	Terminal deletion of distal 21q
	Interphase	Metaphase						
1	4–5	4	**10x1**, 21x1, 21x1, **20x1**	21	4	3	L	Y
2	4	4	21x2, 21x1, **ring(8)x1**	8, 13,19, 21	4	5	L	N
3	3	3	21x1, 21x2	ND	10	21	H	Y*
4	5	5	21x1, 21x3, **Xx1**	21, X	8	8	L	Y
5	4–5	4	21x1, 21x1, **der(5,13)x2**	5, 13, 21	5	18	H	N*
6	5–8	5	**18x1, 22x1**, 21x1, 21;**22x1**, **ring(13)x1**	13, 20, 21, 22, X	8	22	H	Y
7	4	ND	ND	ND	4	22	H	Y*
8	7	7	21x2, 21x2, 21x2, 21x1	ND	7	6	L	Y
9	4	4	21x1, 21x3	ND	5	8	L	Y
10	3	3	21x1, ring(21)x2	ND	5	7	L	Y
11	4	4	21x1, 21x3	ND	4	7	L	Y*
12	6	6	21x1, 21x1, 21x2, 21x2	ND	6	5	L	N*
13	5	5	21x1, 21x1, 21x3	ND	6	5	L	Y
14	4	4	**7x1**, 21x1, 21x1, **markerx1**	ND	4	5	L	N*
**+ Control for typical iAMP21**	**≥5**	**≥5**	**21, 21x4**		**Median: 5**	**Median: 7**	**L: 10/14 (71.4%)** **H: 4/14 (28.6%)**	**Y: 10/14 (71.4%)** **N: 4/14 (28.6%)**

Bold values significes RUNX1 signal is on a chromosome other than chromosome 21.

### 3.3 Genomic complexity of unusual iAMP21‐ALL

**FIGURE 2:**
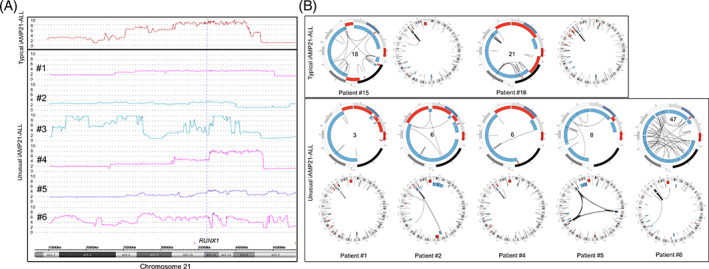
Chromosomal microarray studies reveal copy number fluctuations of chromosome 21 in unusual iAMP21‐ALL cases. (A) Representative chromosome microarray copy number variations analysis using ChAS 3.3 software reveals stepwise gains and losses observed in the smooth signal of typical iAMP21‐ALL. Uneven copy state changes of chromosome 21 is observed in 5 representative cases of unusual iAMP21‐ALL. There is variation in the degree of amplification present in these 5 cases. The vertical blue dotted line represents the genomic location of *RUNX1*. (B) The first row displays MPSeq data for two typical iAMP21‐ALL cases. The first circos plot for each patient shows junctions within chromosome 21 and the second plot is a whole genome view. For the unusual iAMP21‐ALL group, the circos plots at the top represent junctions within chromosome 21 and the bottom circos plots display a whole genome view. The number in the center of the circos plots for chromosome 21 indicate the number of junctions within chromosome 21

CMA studies demonstrated a fluctuating copy number profile of chromosome 21 in all unusual iAMP21‐ALL cases similar in some cases, but in others less pronounced, in comparison to typical iAMP21‐ALL (Figure 2A and data not shown). The median copy number state of chromosome 21 was five in cases classified as unusual iAMP21‐ALL and the range of gains along chromosome 21 varied with some demonstrating gains of up to 10 copies of regions of chromosome 21, as observed in patient 3, while others showed lower‐level gains of four copies of chromosome 21 regions in patients 1, 2, 7, 11, and 14 (Table 2). The median number of fluctuating copy state changes throughout chromosome 21 was 7. The highest number of fluctuating copy state changes was found in four cases (3, 5, 6, and 7). Terminal deletions of distal 21q were also identified in 71% (10/14) of unusual cases and interstitial deletions of variable size of 21q were identified in 43% (6/14) of unusual cases (Figure 2A, Table 2). *RUNX1* was not always located within the region of highest gain. In patient 3, interphase and metaphase FISH identified three copies of *RUNX1*, while CMA confirmed iAMP21‐ALL without amplification of *RUNX1* (Figure 2A). Metaphase FISH targeting 21q22.13 (CTD‐2226O4) and 21q22.3 (CTD‐2119D4) confirmed iAMP21‐ALL with deletion of distal 21q (Figure 1E).

To further characterize the genomic complexity of the unusual iAMP21‐ALL, we performed mate‐pair sequencing (MPseq) on seven cases (5 unusual iAMP21‐ALL and 2 typical iAMP21‐ALL), focusing on those cases with evidence of a *RUNX1* signal observed on a chromosome other than chromosome 21 (Figure 2B). Sequencing data were analyzed for the detection of breakpoint junctions, which is defined as the location of two novel chromosomal breakpoints now joined, and graphically illustrated as previously described.^15^, ^18^ In contrast to 2 typical iAMP21‐ALL cases, which had 18 and 21 junctions within chromosome 21 (patients 15 and 16, respectively), 4 of the 5 unusual cases had a low number of junctions (3–8) within chromosome 21, while one case (patient 6) had a high number of junctions within chromosome 21 consistent with a high number of copy state changes observed by chromosomal microarray (Figure 2A, Table 2). Of the 5 unusual iAMP21‐ALL cases sequenced, rearrangements between chromosome 21 and additional chromosomes including chromosomes 5, 8, 13, 19, 20, 22, and X were identified (Figure 2, Table 2). In addition, 3 of 5 unusual cases had a junction involving the *ERG* gene at 21q22.2, another gene commonly found within the region of amplification in iAMP21‐ALL.

## 4 DISCUSSION

**FIGURE 3:**
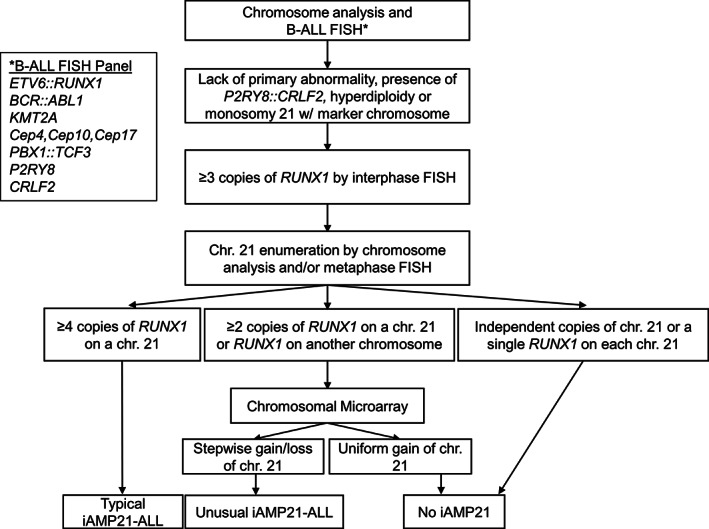
Decision flow chart to aid in the accurate identification of iAMP21‐ALL. An algorithmic approach to utilizing interphase and metaphase B‐ALL FISH and chromosomal microarray to help guide the identification of potential unusual iAMP21‐ALL cases

An *ETV6::RUNX1* probe set has been historically used in the evaluation of B‐ALL patients to identify both *ETV6::RUNX1* fusion associated with *t*(12;21)(p13;q22) and amplification of *RUNX1* associated with iAMP21‐ALL. Here, we demonstrate that approximately 9% of iAMP21‐ALL (19 of 207) in this study cohort did not meet the current definition of iAMP21‐ALL using this probe set. All 14 unusual iAMP21‐ALL cases described in this study would not have been classified as iAMP21‐ALL based only on FISH data alone either because the *RUNX1* FISH signals were located on other chromosomes besides chromosome 21 or they did not have the required greater than or equal to three additional *RUNX1* signals on a single abnormal chromosome 21. The iAMP21‐chromosome was confirmed in these unusual cases using CMA, which revealed a profile of fluctuating copy gains and losses of chromosome 21 (rather than a uniform copy number gain of chromosome 21 seen in the context of high hyperdiploidy). Thus, 9% of iAMP21‐ALL cases would have been missed as having iAMP21‐ALL without the use of CMA. Differentiation between high‐risk iAMP21‐ALL from low‐risk high hyperdiploidy or other B‐ALL genetic subtypes is critical as these entities are associated with different therapeutic approaches and prognostic outlooks. More specifically, patients with iAMP21 treated with standard‐risk (SR) therapy are associated with significantly inferior outcomes and are now recommended to be treated with high‐risk (HR) post‐induction therapy regardless of NCI risk group.^19^ Therefore, in cases with a suspicion of iAMP21‐ALL based‐on cytogenetics and FISH results, CMA should be considered to identify unusual iAMP21‐ALL cases (Figure 3).

Acquired primary genetic abnormalities provide important diagnostic and prognostic information that is essential for risk stratification and treatment selection for patients with ALL. The most recent WHO guidelines include nine recurrent genetic abnormalities, with iAMP21‐ALL listed as a provisional entity.^20^ Most iAMP21‐ALL patients do not have other co‐existing recurrent driver abnormalities; however, a small number of patients have demonstrated co‐occurrence with *P2RY8*::*CRLF2, BCR*::*ABL1*, *ETV6*::*RUNX1* rearrangements or high hyperdiploidy.^3^ In our cohort, one unusual iAMP21‐ALL case had a *P2RY8*::*CRLF2* rearrangement. Patients with iAMP21‐ALL often display secondary genetic abnormalities which include gain of chromosomes X, 10 or 14, or abnormalities involving chromosome 7, deletions of 11q, deletions of *ETV6* and *RB1* and 12q abnormalities involving *SH2B3*.^3^, ^21^, ^22^ Most (12/14) of our unusual iAMP21‐ALL cases displayed at least one of these secondary genetic abnormalities and 3 cases had more than one secondary genetic abnormality. Gain of chromosome X was observed in half of our unusual iAMP21‐ALL cases. Of interest, patient 4 had der(X)t(X;21)(p22.1;q22), resulting in a rearrangement between the *ERG* gene on chromosome 21 and the genomic region immediately upstream of *CRLF2* on chromosome X; whether this rearrangement produced aberrant *CRLF2* gene expression is unknown. Three cases had abnormalities involving chromosome 7, four cases had a deletion of *ETV6* and three cases had loss of *RB1*. Although gain of *RUNX1* signals has historically been used to define iAMP21‐ALL, the variable common region of gain of chromosome 21 encompasses over 40 genes including *ERG* ^*9*^ and amplification of *ERG* has been previously described in cases of iAMP21‐ALL.^23^, ^24^, ^25^ *ERG* is a proto‐oncogenic transcription factor that is involved in cell cycle development and regulation. As *RUNX1* does not appear to be the oncogenic driver of iAMP21‐ALL,^7^ additional studies are warranted to evaluate whether *ERG* is a potential driver of iAMP21‐ALL. In all cases in our study, *ERG* was within the region of chromosome gain, with a copy number ranging from 3 to 8.

To our knowledge, this is the first study to report genomic characterization of iAMP21‐ALL using MPseq. MPseq utilizes a specialized library preparation followed by whole‐genome sequencing to identify structural variations and copy number abnormalities at high resolution (<1 kb). The utility of MPseq in resolving complex chromosomal rearrangements in hematologic malignancies compared to FISH panel testing has been previously described.^18^, ^26^, ^27^ MPseq was able to confirm multiple junctions within chromosome 21, demonstrating complexity of this chromosome in both unusual and typical iAMP21‐ALL. In unusual iAMP21‐ALL, we confirmed rearrangements between chromosome 21 and other chromosomes including chromosomes 5, 8, 13, 19, 20, 22, and X. Although some of these rearrangements were confirmed using metaphase FISH, not all rearrangements identified by MPseq and chromosomal analysis, including metaphase FISH were concordant. Whether this is due to subclonal complexity identified by MPseq or incorrect identification of chromosomes by chromosomal analysis due to poor chromosome morphology is unknown. For example, in case 5, MPseq revealed junctions involving chromosomes 5, 13, and 21, consistent with a complex pattern of gains and losses on 5p and 13q observed by CMA, however, the marker chromosome likely composed of material from chromosomes 5, 13, and 21 was not confidently identified by conventional chromosomes analysis. In case 4, MPseq revealed a junction to chromosome X, conventional cytogenetic analysis identified a der(X)t(X;21)(p22.1;q22), with one *RUNX1* signal identified on chromosome X. In this case, all three testing strategies were consistent. Whether the mechanism causing unusual iAMP21‐ALL, including those cases with rearrangements involving other chromosomes, also involves breakage‐fusion‐bridge cycles is also unknown.^5^, ^8^, ^9^, ^28^, ^29^

## 5 CONCLUSION

In summary, we demonstrate that FISH is insufficient for accurate identification of all iAMP21‐ALL cases. We confirm the value of CMA for validation of ambiguous cases, particularly in those lacking a recurrent primary abnormality or those having monosomy 21 (or with a marker or ring chromosome with *RUNX1* signals). Our study demonstrates that misclassification of iAMP21‐ALL may occur because *RUNX1* may not be located within the highest region of chromosome 21 gain and/or the additional *RUNX1* signals are present on chromosomes other than the abnormal chromosome 21. We have proposed an algorithmic approach to aid in the accurate identification of iAMP21‐ALL utilizing interphase and metaphase B‐ALL FISH and CMA to help guide laboratory geneticists in the identification of unusual iAMP21‐ALL (Figure 3). This study is limited by its retrospective nature, small sample size of the unusual iAMP21‐ALL subtype, and absence of patient outcome data. In addition, this study reports a total percent of unusual iAMP21‐ALL cases that may be misclassified; however, this is the combined experience of a single laboratory (Mayo Clinic) and COG, which represents multiple laboratories. Thus, the individual incidences 7 of 33 or 21% for Mayo Clinic and 12 of 174 or 7% for COG differ and this observation may be explained by the variability in laboratory practices and in the sample sizes of the two cohorts (33 vs. 174). Future studies, including larger cohort sizes, are necessary to identify more precisely the incidence of iAMP21‐ALL, including unusual cases, and to evaluate whether more cases without a primary genetic abnormality may harbor yet unidentified cases of unusual iAMP21‐ALL. Further studies are also needed to evaluate the clinical significance of unusual iAMP21‐ALL in comparison to typical iAMP21‐ALL. Incorporation of CMA or utilization of alternative probe sets on chromosome 21 should be considered in cases with an unknown primary abnormality to evaluate the presence of iAMP21‐ALL for accurate risk stratification.

## AUTHOR CONTRIBUTIONS

**Alaa Koleilat**: conceptualization, data curation, formal analysis, investigation, visualization, writing‐original draft, writing‐review and editing; **James B. Smadbeck**: data curation, formal analysis, investigation, software, visualization; **Cinthya J. Zepeda‐Mendoza**: formal analysis, visualization; **Cynthia M. Williamson**, **Beth A. Pitel**, **Crystal L. Golden**: data curation; **Xinjie Xu**: writing‐review & editing; **Patricia T. Greipp**: writing‐review & editing; **Rhett P. Ketterling**: writing‐review & editing; **Nicole L. Hoppman**: writing‐review & editing; **Jess F. Peterson**: writing‐review & editing; **Christine J. Harrison**, **Yassmine M. N. Akkari**, **Karen D. Tsuchiya**, **Mary Shago**: funding acquisition (COG), data curation, methodology, resources, writing‐review & editing**; Linda B. Baughn**: conceptualization, data curation, formal analysis, funding acquisition (Mayo Clinic), investigation, methodology, project administration, resources, supervision, validation, visualization, writing‐review & editing.

## Supporting information


**Figure S1** Retrospective Evaluation of the Mayo Clinic Genomics Database for B‐ALL Cohort Analysis. (A) To determine the frequency of iAMP21‐ALL in the Mayo Clinic B‐ALL cohort, we performed a retrospective evaluation of the Mayo Clinic Genomics database from January 2018 to December 2020 of all pediatric (≤30 years of age) acute leukemia cases with clinical trial (COG) enrollment. Of 1126 cases in the Mayo Clinic cohort, 777 cases were B‐ALL with the following cytogenetic abnormalities: high hyperdiploidy (51‐67 chromosomes) (*n* = 232, 29.9%), ETV6::RUNX1 fusion (*n* = 151, 19.4%), BCR::ABL1‐like (*n* = 83, 10.7%) (defined in the methods), iAMP21‐ALL (*n* = 33, 4.3%) (typical and unusual), BCR::ABL1 fusion (*n* = 32, 4.1%), KMT2A rearrangement (*n* = 28, 3.6%), TCF3::PBX1 fusion (*n* = 23, 3.0%) and hypodiploidy (<45 chromosomes) (*n* = 17, 2.2%). In 178 cases (22.8%), a recurrent primary abnormality could not be identified, and these were categorized as B‐ALL not otherwise specified (NOS). (B) Due to the rarity of the iAMP21‐ALL subtype, the cohort was expanded to include other iAMP21‐ALL cases in the Mayo Clinic genomics database with a CMA within the same study period (no age restriction and not enrolled to a clinical trial) (*n* = 13). Within the iAMP21‐ALL Mayo Clinic cohort with CMA, seven cases were unusual cases and 26 were typical iAMP21‐ALL (Figure 1A). The number of iAMP21‐ALL cases reviewed by COG from August 2018 to June 2021 was 174 cases. Of these, 7% (12/174) were determined to be unusual iAMP21‐ALL.

## Data Availability

The data that support the findings of this study are available from the corresponding author upon reasonable request.
