# Supplementary material for: Characterization of unusual iAMP21 B‐lymphoblastic leukemia (iAMP21‐ALL) from the Mayo Clinic and Children's Oncology Group
Source: Genes Chromosomes Cancer. 2022 Jul 19;61(12):710–9. doi: 10.1002/gcc.23084 (PMC9549522; doi:10.1002/gcc.23084)
Supplement: Supplementary file 1 — Figure S1 Retrospective Evaluation of the Mayo Clinic Genomics Database for B‐ALL Cohort Analysis. (A) To determine the frequency of iAMP21‐ALL in the Mayo Clinic B‐ALL cohort, we performed a retrospective evaluation of the Mayo Clinic Genomics database from January 2018 to December 2020 of all pediatric (≤30 years of age) acute leukemia cases with clinical trial (COG) enrollment. Of 1126 cases in the Mayo Clinic cohort, 777 cases were B‐ALL with the following cytogenetic abnormalities: high hyperdiploidy (51‐67 chromosomes) (n = 232, 29.9%), ETV6::RUNX1 fusion (n = 151, 19.4%), BCR::ABL1‐like (n = 83, 10.7%) (defined in the methods), iAMP21‐ALL (n = 33, 4.3%) (typical and unusual), BCR::ABL1 fusion (n = 32, 4.1%), KMT2A rearrangement (n = 28, 3.6%), TCF3::PBX1 fusion (n = 23, 3.0%) and hypodiploidy (<45 chromosomes) (n = 17, 2.2%). In 178 cases (22.8%), a recurrent primary abnormality could not be identified, and these were categorized as B‐ALL not otherwise specified (NOS). (B) Due to the rarity of the iAMP21‐ALL subtype, the cohort was expanded to include other iAMP21‐ALL cases in the Mayo Clinic genomics database with a CMA within the same study period (no age restriction and not enrolled to a clinical trial) (n = 13). Within the iAMP21‐ALL Mayo Clinic cohort with CMA, seven cases were unusual cases and 26 were typical iAMP21‐ALL (Figure 1A). The number of iAMP21‐ALL cases reviewed by COG from August 2018 to June 2021 was 174 cases. Of these, 7% (12/174) were determined to be unusual iAMP21‐ALL. [file GCC-61-710-s001.docx]

**Supplementary Material**


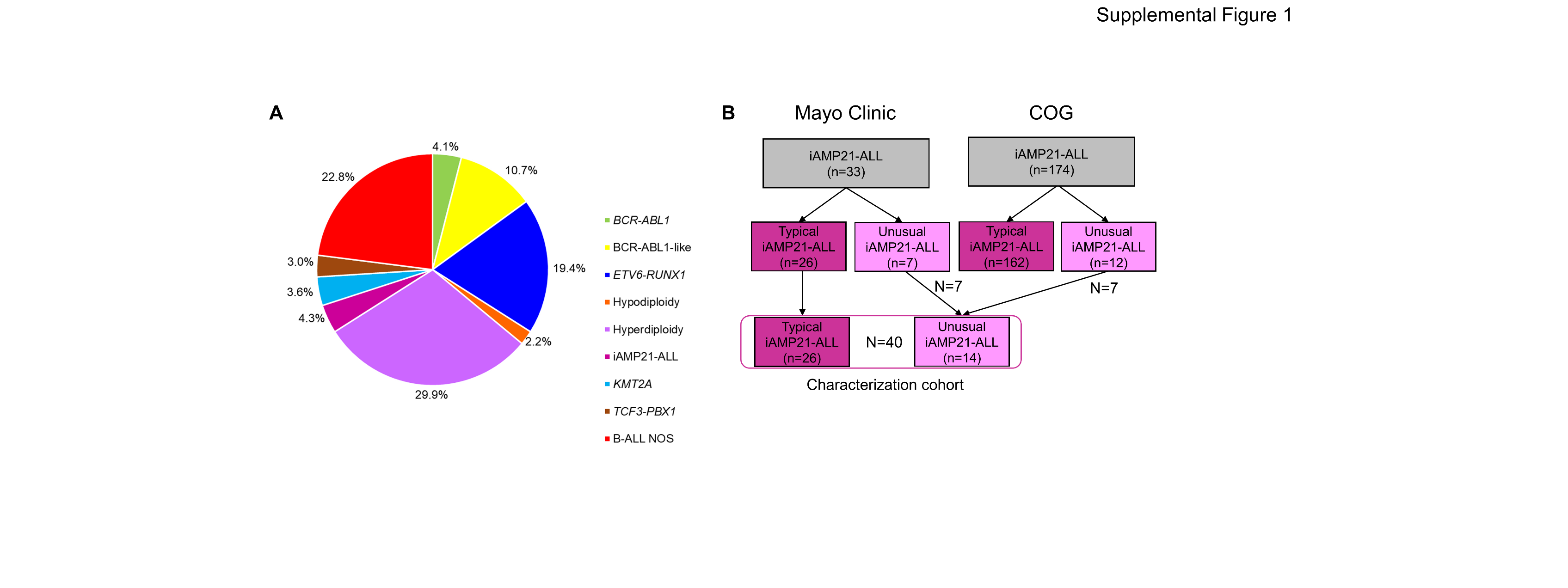


**Supplementary Figure 1: Retrospective Evaluation of the Mayo Clinic Genomics Database for B-ALL Cohort Analysis. A.** To determine the frequency of iAMP21-ALL in the Mayo Clinic B-ALL cohort, we performed a retrospective evaluation of the Mayo Clinic Genomics database from January 2018 to December 2020 of all pediatric (≤30 years of age) acute leukemia cases with clinical trial (COG) enrollment. Of 1126 cases in the Mayo Clinic cohort, 777 cases were B-ALL with the following cytogenetic abnormalities: high hyperdiploidy (51-67 chromosomes) (n=232, 29.9%), *ETV6::RUNX1* fusion (n=151, 19.4%), *BCR::ABL1*-like (n=83, 10.7%) (defined in the methods), iAMP21-ALL (n=33, 4.3%) (typical and unusual), *BCR::ABL1* fusion (n=32, 4.1%), *KMT2A* rearrangement (n=28, 3.6%), *TCF3::PBX1* fusion (n=23, 3.0%) and hypodiploidy (<45 chromosomes) (n=17, 2.2%). In 178 cases (22.8%), a recurrent primary abnormality could not be identified, and these were categorized as B-ALL not otherwise specified (NOS). **B.** Due to the rarity of the iAMP21-ALL subtype, the cohort was expanded to include other iAMP21-ALL cases in the Mayo Clinic genomics database with a CMA within the same study period (no age restriction and not enrolled to a clinical trial) (n=13). Within the iAMP21-ALL Mayo Clinic cohort with CMA, seven cases were unusual cases and 26 were typical iAMP21-ALL (Figure 1A). The number of iAMP21-ALL cases reviewed by COG from August 2018 to June 2021 was 174 cases. Of these, 7% (12/174) were determined to be unusual iAMP21-ALL.
